# SDF-1α is a novel autocrine activator of platelets operating through its receptor CXCR4

**DOI:** 10.1016/j.cellsig.2014.09.021

**Published:** 2015-01

**Authors:** Tony G. Walsh, Matthew T. Harper, Alastair W. Poole

**Affiliations:** School of Physiology and Pharmacology, University of Bristol, Bristol, BS8 1TD, United Kingdom

**Keywords:** SDF-1α, stromal cell-derived factor-1α, CXCR4, CXC chemokine receptor type 4, CXCR7, CXC chemokine receptor type 7, PRP, platelet-rich plasma, TxA_2_, thromboxane A_2_, GPCR, G-protein-coupled receptor, PGE_1_, prostaglandin E_1_, AR-C-66096, AR-C, Platelet, Thrombosis, Signalling, Stromal cell-derived factor-1α, Thromboxane A_2_, Secretion

## Abstract

Platelets store and secrete the chemokine stromal cell-derived factor (SDF)-1α upon platelet activation, but the ability of platelet-derived SDF-1α to signal in an autocrine/paracrine manner mediating functional platelet responses relevant to thrombosis and haemostasis is unknown. We sought to explore the role of platelet-derived SDF-1α and its receptors, CXCR4 and CXCR7 in facilitating platelet activation and determine the mechanism facilitating SDF-1α-mediated regulation of platelet function. Using human washed platelets, CXCR4 inhibition, but not CXCR7 blockade significantly abrogated collagen-mediated platelet aggregation, dense granule secretion and thromboxane (Tx) A_2_ production. Time-dependent release of SDF-1α from collagen-activated platelets supports a functional role for SDF-1α in this regard. Using an in vitro whole blood perfusion assay, collagen-induced thrombus formation was substantially reduced with CXCR4 inhibition. In washed platelets, recombinant SDF-1α in the range of 20–100 ng/mL^− 1^ could significantly enhance platelet aggregation responses to a threshold concentration of collagen. These enhancements were completely dependent on CXCR4, but not CXCR7, which triggered TxA_2_ production and dense granule secretion. Rises in cAMP were significantly blunted by SDF-1α, which could also enhance collagen-mediated Ca(2 +) mobilisation, both of which were mediated by CXCR4. This potentiating effect of SDF-1α primarily required TxA_2_ signalling acting upstream of dense granule secretion, whereas blockade of ADP signalling could only partially attenuate SDF-1α-induced platelet activation. Therefore, this study supports a potentially novel autocrine/paracrine role for platelet-derived SDF-1α during thrombosis and haemostasis, through a predominantly TxA_2_-dependent and ADP-independent pathway.

## Highlights

• Collagen-induced platelet aggregation, TxA_2_ production and dense granule secretion require CXCR4 signalling.
• CXCR4 regulates platelet thrombus formation.
• SDF-1α-induced changes in cAMP and Ca(2 +) signalling require CXCR4.
• SDF-1α, via CXCR4, enhances platelet activation responses to collagen, primarily through a TxA_2_-dependent and ADP-independent pathway.

## 1 Introduction

Platelet activation relevant to thrombosis and haemostasis is a finely coordinated process, involving a complex interplay of subendothelial matrix proteins (collagen, fibrinogen, vWF), localized thrombin and platelet-derived factors, in particular the secondary mediators, ADP and thromboxane (Tx) A_2_, which culminate to regulate platelet signalling responses following vessel damage [1]. Moreover, numerous other bioactive molecules are present in the bloodstream or released from activated platelets, with the ability to potentiate or prime platelet activation and it's believed that these molecules pose a substantial risk to pathological thrombus formation without being significantly relevant to primary haemostasis, thus offering potential therapeutic avenues for targeting thrombotic disorders [2], [3], [4].

Despite being classically known for their chemotactic roles in leukocyte recruitment and inflammatory responses, chemokines represent an additional layer of complexity to the array of known platelet agonists. Platelets express a variety of CC and CXC chemokines and while the majority of these chemokines can support non-conventional aspects of platelet function (i.e. leukocyte recruitment, tissue regeneration) [5], certain chemokines were identified that could induce platelet activation. Macrophage-derived chemokine (MDC, CCL22) and thymus and activation-regulated chemokine (TARC, CCL17) are two CC chemokine receptor 4 (CCR4) ligands, which along with stromal cell-derived factor-1α (SDF-1α, CXCL12) were all shown to induce aggregation in platelet-rich plasma (PRP) and potentiate platelet responses to physiological agonists, ADP and thrombin [6], [7], [8], [9], [10]. More recently, the chemokine's CXCL16 and CX3CL1 (fractalkine) were also demonstrated to trigger platelet activation [11], [12]. Notably, early evidence suggested that platelets did not express these specific chemokines and it was proposed that other vascular sources, including macrophages, endothelial and smooth muscle cells during vasculitis or atherosclerotic plaque rupture, could provide localized concentrations sufficient to potentiate platelet responses [6], [9].

Importantly, evidence in recent years has conclusively shown that platelets express and release functional SDF-1α upon activation, whereas evidence supporting the release of platelet-derived MDC and TARC is less established [13], [14], [15]. Studies have demonstrated novel roles for platelet-derived SDF-1α in recruiting CD34^+^ progenitor cells to arterial thrombi and supporting their differentiation to endothelial progenitor cells in vivo, to facilitate vascular remodelling and repair [16], [17]. More recently, SDF-1α from activated platelets was demonstrated to be crucial for the transformation of circulating monocytes into multipotential cells with the capacity to differentiate into mesenchymal and endothelial lineages [18]. Despite these insights, the possibility of platelet-derived SDF-1α exerting autocrine/paracrine signalling effects regulating platelet activation relevant to thrombosis or haemostasis, in a manner similar to the classical secondary mediators ADP or TxA_2_, has not been explored. Moreover, the mechanisms by which SDF-1α mediates enhancements of platelet function are poorly defined.

SDF-1α was originally believed to signal exclusively via the Gαi-coupled G-protein-coupled receptor (GPCR), CXC chemokine receptor type 4 (CXCR4), but more recent studies identified CXCR7 as a higher affinity receptor for SDF-1α [19], [20], [21], [22]. Like CXCR4, CXCR7 possesses all the canonical components of GPCRs, but it is unable to activate heterotrimeric G proteins, so that the mechanisms mediating activation of intracellular signals remain controversial [23], [24], [25]. CXCR7 is believed to operate as a decoy receptor, which can internalize bound SDF-1α to regulate SDF-1α gradients necessary for optimal signals via CXCR4 [26]. Additionally, both receptors can heterodimerise to regulate SDF-1α-mediated functions [27]. Importantly, both receptors are expressed on the platelet surface and while CXCR4 appears functional, the relevance of CXCR7 to platelet physiology is only starting to be elucidated [6], [28], [29], [30].

With this study, we sought to determine if SDF-1α plays a significant autocrine/paracrine signalling role regulating human platelet function through its two cognate receptors and to address the mechanism of SDF-1α-mediated regulation of platelet activation with relevance to ADP and TxA_2_ signalling. Since the annucleate platelet is not amenable to genetic manipulation, we used a pharmacological inhibitor and blocking antibody approach to delineate novel signalling roles for target proteins. Our findings demonstrate that blocking CXCR4, but not CXCR7, significantly reduced collagen-mediated platelet aggregation, dense granule secretion and TxA_2_ production. Consistently, in vitro collagen-induced thrombus formation required CXCR4-dependent signalling. To support a synergistic signalling mechanism between collagen and SDF-1α, co-stimulation experiments revealed that SDF-1α, via CXCR4, could substantially enhance platelet activation to threshold concentrations of collagen, through an amplification pathway which is primarily TxA_2_-dependent, but ADP-independent.

## 2 Materials and methods

### 2.1 Materials

Platelet agonists used were fibrillar HORM® Collagen (Type I) of equine tendon (Nycomed, Munich, Germany) and human recombinant SDF-1α (R&D Systems Europe, Abingdon, UK). Pharmacological inhibitors; AMD3100 octahydrochloride (CXCR4), MRS-2279 (P2Y_1_), and AR-C66096 (P2Y_12_ — abbreviated AR-C) were from R&D Systems and indomethacin (cyclooxygenase) was from Sigma-Aldrich (Poole, UK). All inhibitors used were dissolved in modified HEPES-Tyrodes, with the exception of indomethacin, which was dissolved in DMSO (vehicle conc. was 0.1%). Anti-CXCR7 blocking antibody (Clone 11G8) and isotype control (Mouse IgG_1_) were from R&D Systems and anti-CD32/FcγRIIA (Clone IV.3) was from StemCell Technologies (Grenoble, France). d-Phenylalanyl-l-propyl-l-arginine chloromethyl ketone (PPACK) was from Calbiochem (Merck Chemicals, Nottingham, UK) and heparin was from Sigma-Aldrich. Unless stated, all other reagents used were from Sigma-Aldrich.

### 2.2 Human platelet preparation

Venous human blood from healthy, drug-free volunteers was drawn in 0.4% trisodium citrate (v/v) in accordance with the local ethics committee and informed consent. In brief, citrated blood was acidified with 12.5% (v/v) acid citrate dextrose (85 mM trisodium citrate, 71 mM citric acid, 111 mM glucose) and centrifuged at 190 ×*g* for 17 min. PRP was removed and centrifuged at 650 ×*g* for 10 min in the presence of 140 nM prostaglandin (PG) E_1_ and 0.02 U mL^− 1^ apyrase (Grade VII, Sigma). The resulting platelet pellet was resuspended at the required density in modified HEPES-Tyrode's buffer (10 mM HEPES, 145 mM NaCL, 1 mM MgCl_2_, 3 mM KCL, 5 mM Glucose, pH 7.3) containing 0.02 U mL^− 1^ apyrase.

### 2.3 Lumi-aggregometry — platelet aggregation and ATP secretion

Simultaneous monitoring of platelet aggregation and ATP secretion was performed at 37 °C with constant stirring (1000 rpm) in a Chronolog 700 aggregometer (Chronolog, Havertown, PA, USA). For all experiments involving pharmacological inhibitors, platelets (2 × 10^8^ mL^− 1^) were pre-treated with vehicle control or inhibitors for 10 min. Also, for experiments involving functional blockade of platelet CXCR7, platelets were pre-incubated for 10 min with 10 μg/mL^− 1^ IV.3 prior to a 10 min incubation with 10 μg/mL^− 1^ anti-CXCR7 (11G8) or IgG_1_ control to prevent non-specific platelet activation via the FcγRIIA receptor. Before agonist stimulation, 5 μL of luciferin-luciferase (Chronolog) was added. Post aggregation, 1 nM ATP standard was added as a reference value for quantification of secreted ATP. Data analysis was performed with Aggro/Link5 software (Chronolog).

### 2.4 Measurement of TxA_2_ production

To assess TxA_2_ levels, the stable metabolite TxB_2_ was analysed using a commercial ELISA kit (Enzo Life Sciences, Exeter, UK). Briefly, activated platelet samples (from aggregation reactions) were quenched at 5 min with 5 mM EDTA and 200 μM indomethacin to inhibit further TxA_2_ formation. Samples were centrifuged for 4 min at 12,000 ×*g*, with the supernatant removed and stored at − 80 °C for subsequent TxB_2_ analysis according to manufacturer's instructions.

### 2.5 SDF-1α ELISA

To monitor soluble SDF-1α release, washed platelets (4 × 10^8^ mL^− 1^) were stimulated under aggregating conditions for indicated times. Samples were immediately transferred to an eppendorf containing 280 nM PGE_1_ (final conc.) and subjected to 2 × pulse centrifugation steps (15 s at 12,000 ×*g*), with the final releasate stored at − 80 °C for subsequent analysis by SDF-1α ELISA as per manufacturer's instructions (R&D Systems).

### 2.6 cAMP ELISA

For quantification of cyclic adenosine monophosphate (cAMP), washed platelets (4 × 10^8^ mL^− 1^) were pre-treated with inhibitors and stimulated with 1 μM PGE_1_ in the absence or presence of SDF-1α for 5 min at 37 °C. cAMP accumulation was terminated by the addition lysis buffer (0.5% TritonX-100) containing 0.1 M HCL to stop endogenous phosphodiesterase activity for 30 min at room temperature. Samples were clarified by centrifugation at 12,000 × *g* for 5 min with the supernatant stored for subsequent analysis by cAMP ELISA according to manufacturer's instructions (Enzo Life Sciences).

### 2.7 Ca(2 +) signalling

Ca(2 +) measurement was performed as previously described [31].

### 2.8 In vitro thrombus formation

Analysis of thrombus formation was performed using an Ibidi® μ-Slide VI^0.1^ flow chamber (Thistle Scientific Ltd, Glasgow, UK) connected to a Harvard Apparatus syringe pump (Kent, UK). Prior to whole blood perfusion, the Ibidi μ-slide channels were coated with 50 μg/mL^− 1^ fibrillar collagen for 2 h at room temperature and blocked in 2% fatty acid free BSA (Sigma) overnight at 4 °C. Channels were flushed with modified HEPES-Tyrodes immediately prior to whole blood perfusion. Anti-coagulated blood drawn in 40 μM PPACK and 2 U mL^− 1^ heparin was loaded with 2 μM DiOC6 (Enzo Life Sciences) in the presence of vehicle control or 100 nM AMD3100 for 10 min. Labelled blood was then perfused at a constant shear rate of 1000 s^− 1^ for 6 min, followed by a 4 min wash/fixation step in modified HEPES-Tyrodes containing 4% para-formaldehyde at a similar shear rate. Images of platelet thrombi were captured through a 40× oil immersion objective on Leica DM IRE2 inverted epifluorescent microscope attached to a Leica TCS-SP2-AOBS confocal LSM. Five randomly chosen fields of view were analysed per sample with a z-axis interval of 1 μm per confocal section. Quantification of surface coverage was performed with ImageJ and thrombus volume using Volocity® 6.1.1 Quantitation software (Perkin Elmer Inc., San Jose, CA, USA).

### 2.9 Statistical analysis

All data was analysed using GraphPad Prism 5 software (GraphPad Software Inc., San Diego, CA, USA). Quantified data are presented as mean ± SEM with the indicated number of independent observations. A value of *P < 0.05 was considered statistically significant and was determined using either a paired Student's t-test or one- and two-way ANOVA with Dunnett's or Bonferroni post hoc test for multiple comparison.

## 3 Results

### 3.1 Collagen-induced SDF-1α release and regulation of platelet aggregation by CXCR4

**Fig. 1:**
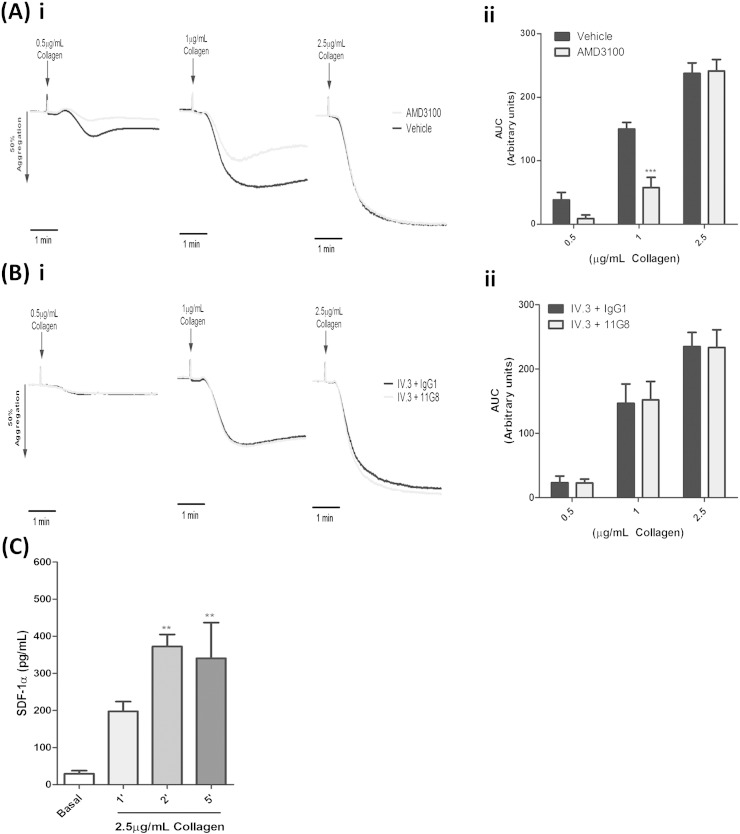
Collagen-induced SDF-1α release and regulation of platelet aggregation by CXCR4. A –B, Washed platelets (2 × 10^8^/mL^− 1^) were pre-treated with vehicle control or inhibitors; (A) 100 nM AMD3100 (CXCR4) and (B) 10 μg/mL^− 1^ 11G8 (CXCR7) or IgG_1_ control in the presence of 10 μg/mL^− 1^ IV.3 and monitored for platelet aggregation using a range of collagen concentrations. Representative aggregation traces (A–Bi) and quantified area under the curve analysis (A–Bii) are shown. Data are mean ± SEM, n = 6, *** P < 0.001 vs. vehicle, Two-way ANOVA with Bonferroni post-hoc analysis. C) Washed platelets (4 × 10^8^ mL^− 1^) were stimulated with 2.5 μg/mL^− 1^ collagen for indicated time points under aggregating conditions, with subsequent analysis of platelet releasate by SDF-1α ELISA. Data are mean ± SEM, n = 4, **P < 0.01 vs. basal, One-way ANOVA with Dunnett's multiple comparison test.

While it is well established that platelets store SDF-1α and exogenously added SDF-1α can induce platelet aggregation in PRP, the possibility of platelet-derived SDF-1α exerting autocrine/paracrine effects on platelet activation is unexplored. To investigate this, we used the pharmacological inhibitor, AMD3100 [32] and blocking antibody 11G8 [33], to target the two known receptors for SDF-1α, CXCR4 and CXCR7, respectively and assessed platelet responses to the physiological agonist collagen. Blocking the CXCR4 receptor yielded a significant reduction in platelet aggregation responses to intermediate but not high collagen doses, as assessed by area under curve analysis (Fig. 1Ai–ii). Targeting CXCR7 with 11G8 did not produce any apparent, functional impairment of aggregation responses across the range of collagen doses (Fig.1Bi–ii). To support a potential role for platelet-derived SDF-1α in regulating CXCR4-mediated regulation of collagen responses, we monitored the release of SDF-1α under similar aggregating conditions to collagen. Analysis of SDF-1α release kinetics by ELISA revealed substantial increases in soluble SDF-1α compared to basal levels, which were statistically significant between 2–5 min post stimulation (Fig. 1C).

### 3.2 Regulation of collagen-induced dense granule secretion and TxA_2_ production by CXCR4

**Fig. 2:**
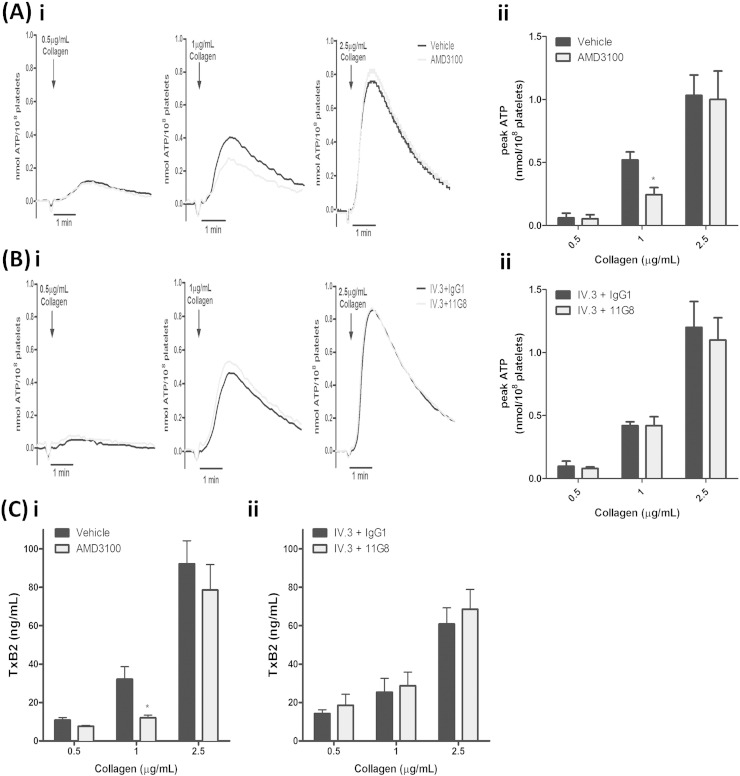
Regulation of collagen-mediated dense granule secretion and TxA_2_ production by CXCR4. Washed platelets (2 × 10^8^/mL^− 1^) were pre-treated with vehicle control or inhibitors; (A, Ci) 100 nM AMD3100 (CXCR4) and (B, Cii) 10 μg/mL^− 1^ 11G8 (CXCR7) or IgG_1_ control in the presence of 10 μg/mL^− 1^ IV.3 and stimulated with increasing concentrations of collagen. ATP release from dense granules (A, B) was assessed by lumi-aggregometry, while TxA_2_ production (C) was monitored by a TxB_2_ ELISA using releasates generated from samples in A–B. Representative secretion traces (A–Bi) and peak ATP release values (nmoL ATP/10^8^ platelets, A–Bii) are shown. Data are mean ± SEM, n = 4, *P < 0.05 vs. vehicle, Two-way ANOVA with Bonferroni post-hoc analysis.

To monitor dense granule secretion, we assessed ATP release by lumi-aggregometry, which demonstrated that CXCR4 inhibition induced a significant reduction in peak ATP secretion values at intermediate collagen concentrations, but not with higher concentrations of collagen (Fig. 2Ai–ii). However, no obvious changes in the rate of ATP secretion were observed. Similarly, blocking CXCR4 leads to a significant reduction in total TxA_2_ levels, which was only apparent at intermediate collagen concentrations (Fig.2). Consistent with the lack of effect on collagen-induced aggregation, blockade of CXCR7 signalling with 11G8 did reveal any significant changes in ATP secretion or TxA_2_ responses to collagen (Fig.2Bi–ii, Cii).

### 3.3 Collagen-induced thrombus formation requires CXCR4-dependent signalling

**Fig. 3:**
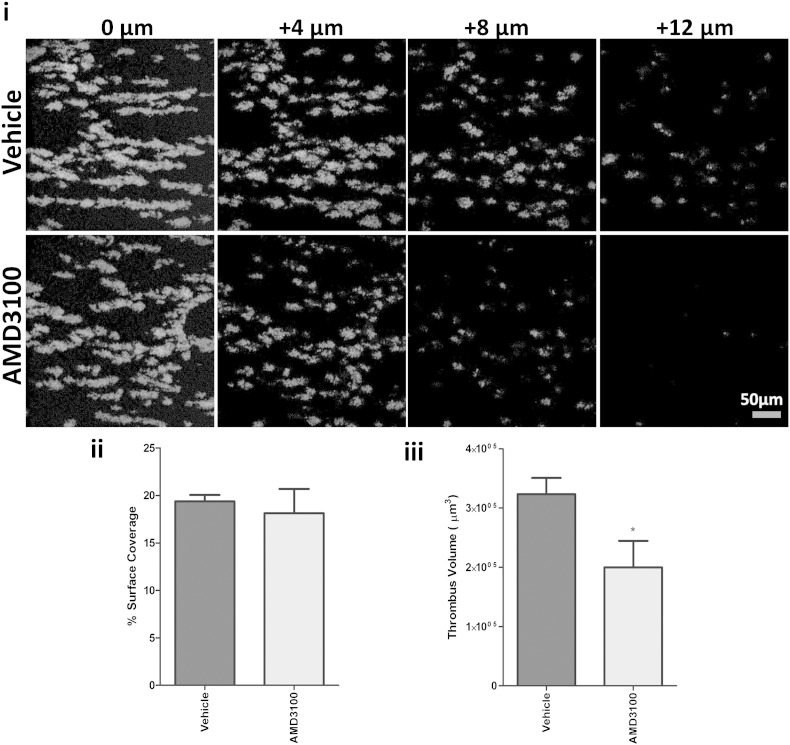
Collagen-induced thrombus formation requires CXCR4-dependent signalling. PPACK- (40 μM) and Heparin- (2 U/mL^− 1^) anticoagulated whole blood labelled with 2 μM DiOC6 was pre-treated with vehicle control or 100 nM AMD3100 and perfused over a collagen-coated surface for 6 min at a constant shear of 1000 s^− 1^. Samples were subsequently washed and fixed in modified HEPES-Tyrodes containing 4% paraformaldehyde. Representative confocal images at the collagen surface (z position = 0 μm) with 4 μm increments, as indicated, are shown along with quantitative analysis of platelet thrombus coverage (%) and volume (μm^3^) (Ai–iii). Data are mean ± SEM, n = 4, *P < 0.05 vs. vehicle, two-tailed paired Student's t-test.

Considering the defect in collagen-induced platelet activation with CXCR4 inhibition, we sought to investigate the role for this receptor on thrombus formation over collagen at arterial shear. We utilized confocal microscopy to facilitate assessment of thrombus volume and thrombus coverage at the collagen surface. Notably, there was no significant difference in percentage platelet coverage of the collagen surface (i.e. 0 μm z-position) between vehicle and AMD3100 treated samples (Fig. 3ii). However, as shown by representative end-point images at different z planes and the subsequent analysis of thrombus volume, inhibition of CXCR4 significantly reduced thrombus accumulation (Fig. 3i, iii). The data suggest that CXCR4 contributes to the thrombus build up, but not to the initial adhesion of platelets to the collagen-coated surface.

### 3.4 SDF-1α-mediated enhancement of platelet activation by CXCR4

**Fig. 4:**
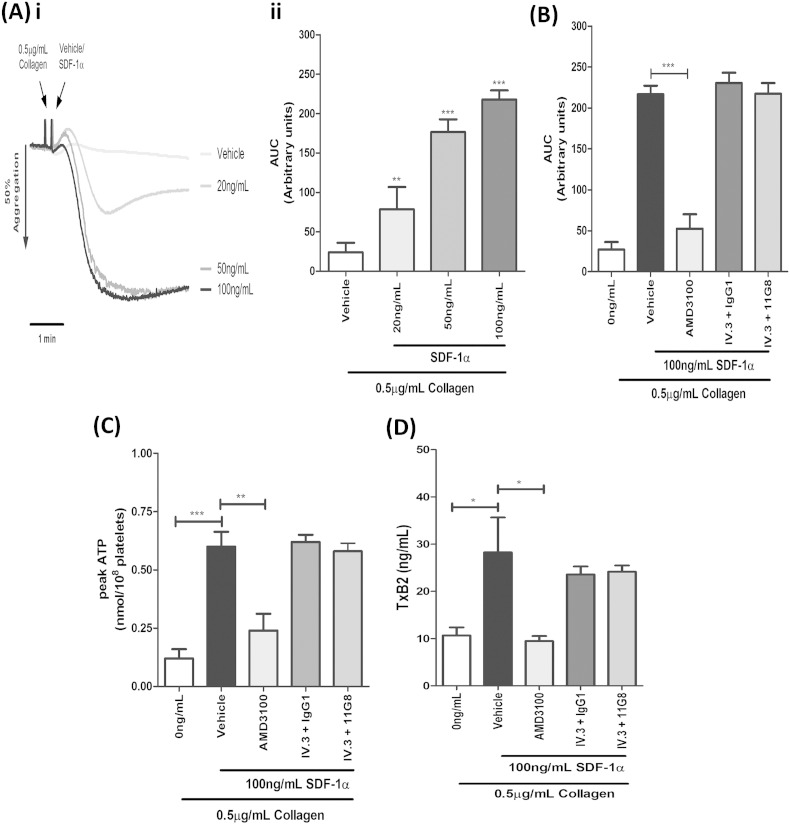
SDF-1α-mediated enhancement of platelet activation by CXCR4. A, Washed platelets (2 × 10^8^/mL^− 1^) were co-stimulated with a threshold concentration of collagen (0.5 μg/mL^− 1^) and increasing concentrations of SDF-1α, and monitored for platelet aggregation. A representative trace (Ai) and quantified area under the curve analysis (Aii) are shown. Data are mean ± SEM, n = 10, **P < 0.01, ***P < 0.001 vs vehicle, One-way ANOVA with Dunnett's multiple comparison test. B–D). Similarly, washed platelets (2 × 10^8^/mL^− 1^) were pre-treated with vehicle control or inhibitors; 100 nM AMD3100 (CXCR4) and 10 μg/mL^− 1^ 11G8 (CXCR7) or IgG_1_ control in the presence of 10 μg/mL^− 1^ IV.3 and co-stimulated with 0.5 μg/mL^− 1^ collagen and 100 ng/mL^− 1^ SDF-1α. Platelet aggregation with area under the curve analysis (B) and peak ATP secretion (C) were assessed by lumi-aggregometry, with the releasates from aggregated samples analysed for TxB_2_ (D). Data are mean ± SEM, n = 4 (n = 6 for B), *P < 0.05, **P < 0.01, ***P < 0.001, One-way ANOVA with Dunnett's multiple comparison test.

SDF-1α alone did not induce platelet activation in washed platelets (data not shown), which is comparable with previous reports and studies using agonists targeting other Gαi-coupled receptors [9], [34]. To address enhancements of platelet function attributable to SDF-1α in a washed platelet system, platelets were co-stimulated with a threshold concentration of collagen and recombinant SDF-1α, which would also allow further investigation into the relative roles for CXCR4 and CXCR7 in this regard. SDF-1α dose-dependently enhanced the aggregation response to 0.5 μg/mL^− 1^ collagen, with significant enhancements observed within the range of 20–100 ng/mL^− 1^ SDF-1α (Fig. 4Ai–ii). Subsequent experiments confirmed that SDF-1α-mediated enhancements of platelet aggregation (Fig. 4B), dense granule secretion (Fig. 4C) and TxA_2_ production (Fig. 4D) in the presence of low dose collagen, were significantly attenuated by pre-treatment with the CXCR4 inhibitor AMD3100, whereas blocking CXCR7 did not affect any of these responses.

### 3.5 Regulation of cAMP and Ca(2 +) signalling by SDF-1α

**Fig. 5:**
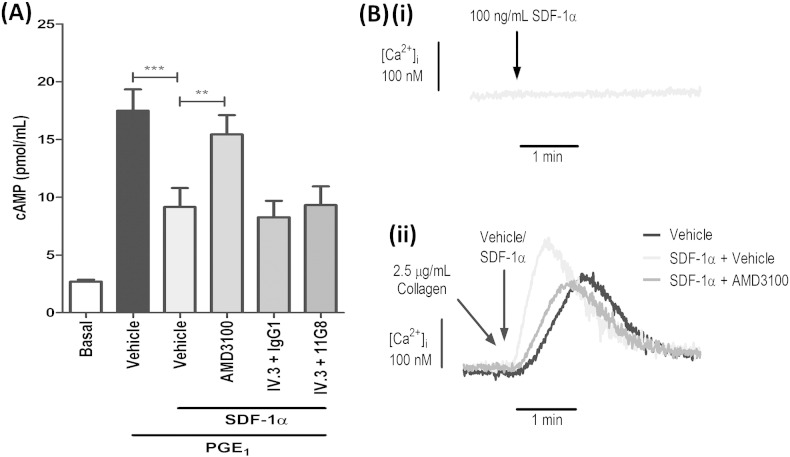
Regulation of cAMP and Ca(2 +) signalling by SDF-1α. A, Washed platelets (4 × 10^8^/mL^− 1^) were pre-treated with vehicle control or 100 nM AMD3100 and then stimulated with 0.7% EtoH (final conc. — carrier for PGE_1_) or 1 μM PGE_1_ in the absence or presence of 100 ng/mL^− 1^ SDF-1α. Samples were lysed and analyzed for total cAMP levels (pmol/mL^− 1^) by ELISA. Data are mean ± SEM, n = 4, **P < 0.01, ***P < 0.001, One-way ANOVA with Dunnett's multiple comparison test. B, Washed platelets (1 × 10^8^/mL^− 1^) were loaded with Fura2-AM and stimulated with 100 ng/mL^− 1^ SDF-1α alone (Bi) or co-stimulated with 2.5 μg/mL^− 1^ collagen and 100 ng/mL^− 1^ SDF-1α (or vehicle) in the absence or presence of 100 nM AMD3100 to monitor changes in intracellular calcium responses (Bii). Traces shown are representative of 4 independent experiments.

Seeing as both CXCR4 and CXCR7 are reported to be Gαi-coupled GPCRs, we sought to investigate the role of each receptor on cAMP levels in response to SDF-1α stimulation. Similar to the findings by Kowalska et al. [9], SDF-1α could significantly reduce the rise in cAMP evoked by PGE_1_ stimulation of prostaglandin receptors. Moreover, this effect was significantly inhibited by CXCR4 inhibition (Fig. 5A), while blocking CXCR7 had no effect (Fig. 5A). Importantly, blockade of the Gi-coupled P2Y_12_ receptor with AR-C did not interfere with SDF-1α-mediated changes in cAMP, ruling out the possibility of secreted ADP facilitating this response (data not shown). Discrepancies regarding the ability of SDF-1α to evoke rises in intracellular Ca(2 +) exist [6], [8]. In our hands, SDF-1α alone could not induce alterations in cytosolic Ca(2 +)_,_ but when co-stimulated with collagen, it produced a profound rise in the rate of the Ca(2 +) signal that was substantially attenuated by CXCR4 inhibition (Fig. 5Bi–ii).

### 3.6 SDF-1α-mediated enhancement of platelet activation is primarily TxA_2_-dependent and ADP-independent

**Fig. 6:**
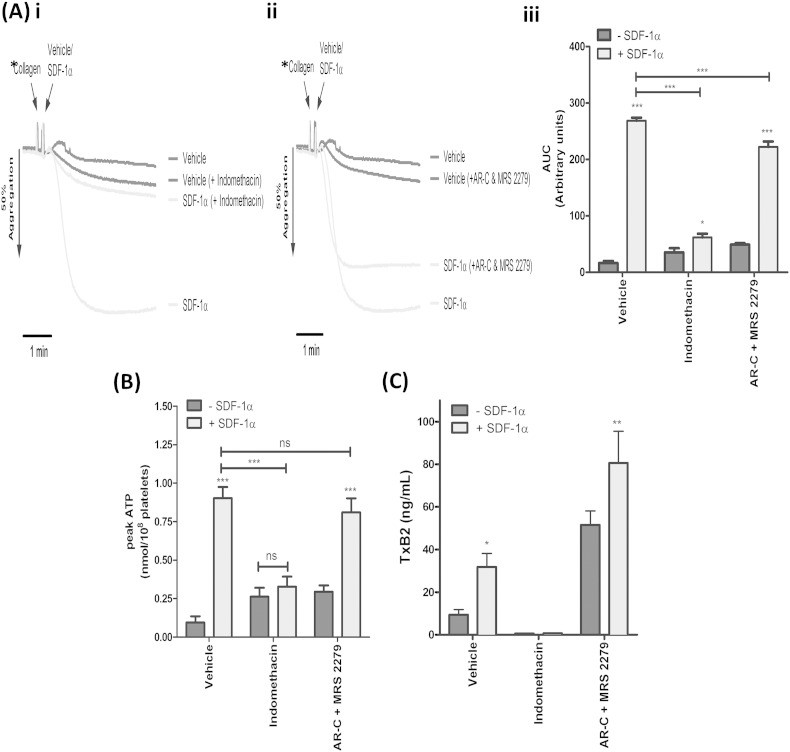
SDF-1α-mediated enhancement of platelet activation is primarily TxA_2_-dependent and ADP-independent. Washed platelets (2 × 10^8^/mL^− 1^) were pre-treated with vehicle control or inhibitors; 10 μM indomethacin (cyclooxygenase) or 1 μM AR-C and 10 μM MRS 2279 (P2Y_1_ and P2Y_12_, respectively) and co-stimulated with threshold doses of collagen and 100 ng/mL^− 1^ SDF-1α. For inhibitor treated samples, platelets were stimulated with 2 μg/mL^− 1^ collagen (vs. 0.5 μg/mL^− 1^ in non-inhibitor treated samples) to induce threshold aggregation responses that were comparable to non-inhibitor treated samples. * in Ai–ii denotes different collagen concentrations. Platelet aggregation with representative traces (Ai–ii), area under the curve analysis (Aiii) and peak ATP secretion (B) were assessed by lumi-aggregometry, with the releasates from aggregated samples analysed for TxB_2_ (C). Data are mean ± SEM, n = 5, non-significant (ns), *P < 0.05, **P < 0.01, ***P < 0.001 vs. vehicle, Two-way ANOVA with Bonferroni post-hoc analysis.

To explore the mechanism(s) mediating SDF-1α-induced enhancements of platelet activation, platelets were pre-treated with or without indomethacin or the ADP receptor antagonists, AR-C and MRS 2279, to ablate TxA_2_- and ADP-dependent signalling, respectively, and then co-stimulated with threshold concentrations of collagen and 100 ng/mL^− 1^ SDF-1α. Blocking TxA_2_ signalling with indomethacin (Fig. 6C) substantially abrogated the enhanced aggregation and ATP secretion responses to SDF-1α, although the aggregation response was not completely suppressed (Fig. 6Ai, iii, B). Inhibition of ADP signalling revealed that SDF-1α could still induce significant enhancements of platelet aggregation (Fig. 6Aii–iii), ATP secretion (Fig. 6B) and TxA_2_ production (Fig. 6C), although aggregation responses were not fully recovered (Fig. 6Aiii).

## 4 Discussion

It is clear that there are multiple mechanisms controlling platelet function which dictates the extent of the response necessary to control bleeding or facilitate thrombus growth leading to coronary and cerebrovascular ischemic events. Currently, the most prevalent anti-platelet therapies prophylactically administered to cardiovascular disease risk patients involve targeting TxA_2_ and ADP, via aspirin and clopidogrel, respectively. Despite proven effectiveness, these antiplatelet agents have experienced issues of non-responsiveness, while attempts to induce more potent inhibition of platelet function have been associated with bleeding complications [35], [36], [37]. With this study, we aimed to explore the role of platelet-derived SDF-1α as a potentially novel, autocrine/paracrine mediator of platelet function in response to the primary platelet agonist collagen and to determine the mechanism through which SDF-1α exerts its enhancing effects on platelet activation.

To exclude a potential role for plasma-derived SDF-1α, our initial studies in washed platelets revealed an important role for the SDF-1α receptor, CXCR4, but not CXCR7 in regulating platelet aggregation and activation responses to intermediate concentrations of collagen. Notably, TxA_2_ production and dense granule secretion were significantly reduced by CXCR4 inhibition in response to intermediate collagen doses, providing a mechanistic basis for the defect in platelet aggregation. Indeed, platelet responses to collagen are primarily dependent on signalling via secondary mediators, TxA_2_ and ADP [38]. To support a role for SDF-1α in this regard, we demonstrate the time-dependent release of SDF-1α release under similar aggregating conditions, in line with previous studies which show SDF-1α release under different conditions [16], [29]. Moreover, in a separate experiment, it was observed that only 36% of recombinant SDF-1α could be recovered from a platelet suspension when compared to a buffer alone sample, suggesting that the ELISA values somewhat underestimate the actual released values for SDF-1α (data not shown). Nonetheless, this supports a model that SDF-1α released from activated platelets can act back on itself, consistent with the assertion that SDF-1α has a strong affinity to remain surface bound under both resting and activated conditions [39].

Considering the defect in platelet responses to collagen following CXCR4 inhibition, we expanded our observations to an in vitro whole blood perfusion assay over a collagen-coated surface to monitor thrombus formation at arterial shear. Two-dimensional end-point analysis of platelet deposition at the collagen surface revealed no significant difference in thrombus coverage, but subsequent three-dimensional analysis of thrombus volume revealed a significant reduction in the presence of the CXCR4 inhibitor, AMD3100. These observations support a model that the initial phase of platelet adhesion to collagen is preserved, but the secondary, expansion phase involving the recruitment of additional platelets to the growing thrombus is abrogated, which is similar to the thrombus phenotype observed in the presence of aspirin or P2Y_12_ inhibitors [40].

Our evidence supports a functional role for the CXCR4 receptor in the secondary phase of platelet activation. However, despite the initial belief that SDF-1α and CXCR4 were a monogamous ligand-receptor pair, subsequent studies identified alternative ligands for CXCR4. The ubiquitously expressed chemokine, macrophage migration inhibition factor (MIF) was demonstrated as a noncognate ligand of CXCR4 (and CXCR2), shown to facilitate leukocyte recruitment during inflammatory diseases such as atherosclerosis [41]. Currently, there is no evidence to suggest that MIF can act as a platelet agonist. On the contrary, bile salt-dependent lipase (BSDL), which contains structural homology to the V3 loop of HIV-1 that also binds CXCR4, was shown to regulate platelet activation via CXCR4 and accumulate at vessel injury sites in a CXCR4-dependent manner [42]. Moreover, mice deficient in SDF-1α (or CXCR4) die perinatally due to numerous development defects excluding an experimental approach involving SDF-1α deficient platelets [43]. This ambiguous nature of CXCR4 ligands makes accurate inferences about their precise roles under physiological conditions more challenging and we therefore phrase our findings in terms of a ‘potential’ role for platelet-derived SDF-1α as an autocrine/paracrine mediator of platelet function.

To address these limitations, we designed experiments involving threshold concentrations of collagen and exogenously added SDF-1α to examine the synergistic contributions of CXCR4 or CXCR7 to SDF-1α-mediated enhancements of platelet activation. SDF-1α alone did not induce an aggregation response in washed platelets (data not shown). However, in combination with low-dose collagen, SDF-1α could elicit substantial enhancements in platelet aggregation responses, which were statistically significant at doses as low as 20 ng/mL^− 1^ and maximally so within the range of 50–100 ng/mL^− 1^. This SDF-1α-mediated enhancement of aggregation was almost completely suppressed with CXCR4 inhibition, whereas blockade of CXCR7 elicited no apparent effect. Furthermore, elevated TxA_2_ levels and dense granule secretion were observed under these conditions, both of which were regulated by CXCR4.

Gαi-coupled GPCRs signal to inhibit adenylyl cyclase and thereby downregulate cAMP levels, which is associated with enhanced platelet aggregation and platelet accumulation in vivo [44], [45]. Our investigations demonstrate that SDF-1α could significantly attenuate the rise in cAMP induced by PGE_1_, which is consistent with a previous study [9]. Our observations confirm that this inhibitory effect is mediated by CXCR4, whereas CXCR7 blockade had no effect. This was consistent with the literature that CXCR7 cannot induce heterotrimeric G-protein activity and does not couple to signalling events [23]. cAMP is known to modulate platelet function by interfering with both Ca(2 +) entry and intracellular Ca(2 +) mobilisation, which is critical for platelet activation during thrombosis and haemostasis [46], [47]. In our hands, SDF-1α alone did not induce intracellular Ca(2 +) signals, but co-stimulation with collagen facilitated a rapid, enhanced rate of Ca(2 +) mobilisation, which was substantially ablated with CXCR4 inhibition. These elevated Ca(2 +) signals are critical for activating various sensors and key regulatory enzymes, including the Ca(2 +)-dependent cytosolic phospholipase A_2_ (cPLA_2_), which is essential for TxA_2_ production through mobilisation of arachidonic acid [48], thereby providing a possible signalling node through which SDF-1α amplifies TxA_2_ production.

Notably, we did not observe any functional detriment to platelet-collagen responses by interfering with CXCR7 function. These results are not entirely surprising considering that CXCR7 does not activate Gαi-driven pathways and Ca(2 +) mobilisation, unlike CXCR4 [24], [49]. Interestingly, Chatterjee and colleagues recently demonstrated enhanced platelet surface levels of CXCR7 following SDF-1α stimulation, which reduced agonist-induced apoptosis [29]. Since SDF-1α is known to mediate pro-survival signals, these data suggest a non-conventional role for CXCR7 in platelets supporting potential regenerative and repair mechanisms following vessel injury, which correlates with clinical evidence showing enhanced platelet CXCR7 levels in patients with myocardial infarction [28], [50].

The augmented TxA_2_ and dense granule secretion responses to collagen in the presence of SDF-1α alluded to a key role for TxA_2_ or ADP as secondary mediators of the response to SDF-1α. Subsequently, we investigated the ability of SDF-1α to enhance platelet aggregation to collagen in the presence of a cyclooxygenase inhibitor (indomethacin) or ADP receptor antagonists, P2Y_1_ (MRS 2279) and P2Y_12_ (AR-C). Blocking TxA_2_ production with indomethacin significantly attenuated the SDF-1α-induced enhancements of platelet aggregation and ATP secretion, which also imply that TxA_2_ generation is upstream of dense granule secretion. However, in the absence of TxA_2_, SDF-1α still exhibited a weak, yet statistically significant enhancement of platelet aggregation compared to non SDF-1α-treated samples, suggesting a partial TxA_2_-independent regulation of platelet activation. This finding supports previous observations demonstrating that SDF-1α induced platelet aggregation in PRP consists of a primary, TxA_2_-independent wave of aggregation, followed by a secondary TxA_2_-dependent wave [6]. Surprisingly, in the presence of P2Y_1_ and P2Y_12_ receptor antagonists, SDF-1α could still induce profound, statistically significant enhancements of aggregation, ATP secretion and TxA_2_ production. However, aggregation responses to SDF-1α were not fully recovered in the absence of ADP signalling implying a subtle, yet functional role for dense granule secretion facilitating platelet activation by SDF-1α. Overall, these findings implicate a predominantly TxA_2_-dependent and ADP-independent signalling pathway driving SDF-1α-mediated responses. Interestingly though, these observations are in contrast to a study by Borst et al., which revealed that CXCL16-induced platelet activation is ADP-dependent and TxA_2_-independent, alluding to divergent signalling networks propagated by chemokines in platelets [11].

From a clinical context, our data highlights potential drawbacks in currently prescribed anti-platelet therapies, particularly with respect to P2Y_12_ antagonism. In vivo studies have clearly demonstrated that SDF-1α is highly abundant in coronary thrombi during the acute phase following vessel injury, providing localized concentrations that not only facilitate progenitor cell recruitment, but may also potentiate platelet activation responses [16]. Similarly, platelet surface expression of SDF-1α is enhanced in patients with acute coronary syndrome and reduced systolic ventricular function [51]. Also pertinent, several studies have documented elevated plasma SDF-1α levels in different cancerous and inflammatory patient populations [52], [53]. Most striking was the observation that elevated SDF-1α levels (mean value of 10.8 ng/mL^− 1^) were the single predictor of ischemic stroke events when compared to traditional risk factors such as hypertension, dyslipidemia and tobacco use [54]. Such continuous exposure of platelets to elevated levels of plasma SDF-1α, in conjunction with other blood borne factors would facilitate a priming effect on platelets, thereby exacerbating platelet responses to subsequent stimuli [2].

## 5 Conclusion

Our study implicates a potentially novel autocrine/paracrine signalling role for platelet-derived SDF-1α acting exclusively via CXCR4, culminating in enhanced TxA_2_ production and dense granule secretion to facilitate thrombus formation. The platelet enhancing effects of SDF-1α to collagen are primarily driven by TxA_2_-dependent signalling, whereas ADP-mediated signalling appears to contribute minimally. Importantly, we provide sufficient evidence to support a more prominent role for platelet-released SDF-1α in the array of platelet activation events critical for thrombosis and haemostasis.

## Conflict of interest

The authors have no conflicts of interest.

## Contributors

T.G. Walsh performed experiments, interpreted results and wrote the manuscript. M.T. Harper performed experiments, interpreted results and revised the manuscript. A.W. Poole designed research, interpreted results and revised the manuscript.

## References

[1] Broos K., Feys H.B., De Meyer S.F., Vanhoorelbeke K., Deckmyn H. (2011). Blood Rev..

[2] Gresele P., Falcinelli E., Momi S. (2008). Trends Pharmacol. Sci..

[3] Hers I. (2007). Blood.

[4] Pasquet J.M., Gross B.S., Gratacap M.P., Quek L., Pasquet S., Payrastre B., van Willigen G., Mountford J.C., Watson S.P. (2000). Blood.

[5] Gleissner C.A., von Hundelshausen P., Ley K. (2008). Arterioscler. Thromb. Vasc. Biol..

[6] Abi-Younes S., Sauty A., Mach F., Sukhova G.K., Libby P., Luster A.D. (2000). Circ. Res..

[7] Abi-Younes S., Si-Tahar M., Luster A.D. (2001). Thromb. Res..

[8] Gear A.R., Suttitanamongkol S., Viisoreanu D., Polanowska-Grabowska R.K., Raha S., Camerini D. (2001). Blood.

[9] Kowalska M.A., Ratajczak M.Z., Majka M., Jin J., Kunapuli S., Brass L., Poncz M. (2000). Blood.

[10] Shenkman B., Brill A., Brill G., Lider O., Savion N., Varon D. (2004). J. Thromb. Haemost..

[11] Borst O., Munzer P., Gatidis S., Schmidt E.M., Schonberger T., Schmid E., Towhid S.T., Stellos K., Seizer P., May A.E., Lang F., Gawaz M. (2012). Circ. Res..

[12] Schafer A., Schulz C., Eigenthaler M., Fraccarollo D., Kobsar A., Gawaz M., Ertl G., Walter U., Bauersachs J. (2004). Blood.

[13] Fujisawa T., Fujisawa R., Kato Y., Nakayama T., Morita A., Katsumata H., Nishimori H., Iguchi K., Kamiya H., Gray P.W., Chantry D., Suzuki R., Yoshie O. (2002). J. Allergy Clin. Immunol..

[14] Chatterjee M., Huang Z., Zhang W., Jiang L., Hultenby K., Zhu L., Hu H., Nilsson G.P., Li N. (2011). Blood.

[15] Huang Z., Rahman M.F., Jiang L., Xie H., Hu H., Lui W.O., Li N. (2012). J. Thromb. Haemost..

[16] Massberg S., Konrad I., Schurzinger K., Lorenz M., Schneider S., Zohlnhoefer D., Hoppe K., Schiemann M., Kennerknecht E., Sauer S., Schulz C., Kerstan S., Rudelius M., Seidl S., Sorge F., Langer H., Peluso M., Goyal P., Vestweber D., Emambokus N.R., Busch D.H., Frampton J., Gawaz M. (2006). J. Exp. Med..

[17] Stellos K., Langer H., Daub K., Schoenberger T., Gauss A., Geisler T., Bigalke B., Mueller I., Schumm M., Schaefer I., Seizer P., Kraemer B.F., Siegel-Axel D., May A.E., Lindemann S., Gawaz M. (2008). Circulation.

[18] Seta N., Okazaki Y., Miyazaki H., Kato T., Kuwana M. (2013). PLoS One.

[19] Bleul C.C., Farzan M., Choe H., Parolin C., Clark-Lewis I., Sodroski J., Springer T.A. (1996). Nature.

[20] Oberlin E., Amara A., Bachelerie F., Bessia C., Virelizier J.L., Arenzana-Seisdedos F., Schwartz O., Heard J.M., Clark-Lewis I., Legler D.F., Loetscher M., Baggiolini M., Moser B. (1996). Nature.

[21] Balabanian K., Lagane B., Infantino S., Chow K.Y., Harriague J., Moepps B., Arenzana-Seisdedos F., Thelen M., Bachelerie F. (2005). J. Biol. Chem..

[22] Burns J.M., Summers B.C., Wang Y., Melikian A., Berahovich R., Miao Z., Penfold M.E., Sunshine M.J., Littman D.R., Kuo C.J., Wei K., McMaster B.E., Wright K., Howard M.C., Schall T.J. (2006). J. Exp. Med..

[23] Rajagopal S., Kim J., Ahn S., Craig S., Lam C.M., Gerard N.P., Gerard C., Lefkowitz R.J. (2010). Proc. Natl. Acad. Sci. U. S. A..

[24] Kumar R., Tripathi V., Ahmad M., Nath N., Mir R.A., Chauhan S.S., Luthra K. (2012). Cell. Immunol..

[25] Singh A.K., Arya R.K., Trivedi A.K., Sanyal S., Baral R., Dormond O., Briscoe D.M., Datta D. (2013). Cytokine Growth Factor Rev..

[26] Naumann U., Cameroni E., Pruenster M., Mahabaleshwar H., Raz E., Zerwes H.G., Rot A., Thelen M. (2010). PLoS One.

[27] Levoye A., Balabanian K., Baleux F., Bachelerie F., Lagane B. (2009). Blood.

[28] Rath D., Chatterjee M., Borst O., Muller K., Stellos K., Mack A.F., Bongartz A., Bigalke B., Langer H., Schwab M., Gawaz M., Geisler T. (2014). Eur. Heart J..

[29] Chatterjee M., Seizer P., Borst O., Schonberger T., Mack A., Geisler T., Langer H.F., May A.E., Vogel S., Lang F., Gawaz M. (2014). FASEB J..

[30] Clemetson K.J., Clemetson J.M., Proudfoot A.E., Power C.A., Baggiolini M., Wells T.N. (2000). Blood.

[31] Harper M.T., Poole A.W. (2013). Cell Death Dis..

[32] Broxmeyer H.E., Orschell C.M., Clapp D.W., Hangoc G., Cooper S., Plett P.A., Liles W.C., Li X., Graham-Evans B., Campbell T.B., Calandra G., Bridger G., Dale D.C., Srour E.F. (2005). J. Exp. Med..

[33] Torossian F., Anginot A., Chabanon A., Clay D., Guerton B., Desterke C., Boutin L., Marullo S., Scott M.G., Lataillade J.J., Le Bousse-Kerdiles M.C. (2014). Blood.

[34] Nash C.A., Severin S., Dawood B.B., Makris M., Mumford A., Wilde J., Senis Y.A., Watson S.P. (2010). J. Thromb. Haemost..

[35] Bhatt D.L. (2007). N. Engl. J. Med..

[36] Snoep J.D., Hovens M.M., Eikenboom J.C., van der Bom J.G., Jukema J.W., Huisman M.V. (2007). Am. Heart J..

[37] Krasopoulos G., Brister S.J., Beattie W.S., Buchanan M.R. (2008). BMJ.

[38] Nieswandt B., Watson S.P. (2003). Blood.

[39] Chatterjee M., Gawaz M. (2013). J. Thromb. Haemost..

[40] Mendolicchio G.L., Zavalloni D., Bacci M., Corrada E., Marconi M., Lodigiani C., Presbitero P., Rota L., Ruggeri Z.M. (2011). J. Thromb. Haemost..

[41] Bernhagen J., Krohn R., Lue H., Gregory J.L., Zernecke A., Koenen R.R., Dewor M., Georgiev I., Schober A., Leng L., Kooistra T., Fingerle-Rowson G., Ghezzi P., Kleemann R., McColl S.R., Bucala R., Hickey M.J., Weber C. (2007). Nat. Med..

[42] Panicot-Dubois L., Thomas G.M., Furie B.C., Furie B., Lombardo D., Dubois C. (2007). J. Clin. Invest..

[43] Ma Q., Jones D., Borghesani P.R., Segal R.A., Nagasawa T., Kishimoto T., Bronson R.T., Springer T.A. (1998). Proc. Natl. Acad. Sci. U. S. A..

[44] Bellem A., Meiyappan S., Romans S., Einstein G. (2011). Gend. Med..

[45] Sim D.S., Merrill-Skoloff G., Furie B.C., Furie B., Flaumenhaft R. (2004). Blood.

[46] Geiger J., Nolte C., Walter U. (1994). Am. J. Physiol..

[47] Varga-Szabo D., Braun A., Nieswandt B. (2009). J. Thromb. Haemost..

[48] Kita Y., Ohto T., Uozumi N., Shimizu T. (2006). Biochim. Biophys. Acta.

[49] Sierro F., Biben C., Martinez-Munoz L., Mellado M., Ransohoff R.M., Li M., Woehl B., Leung H., Groom J., Batten M., Harvey R.P., Martinez A.C., Mackay C.R., Mackay F. (2007). Proc. Natl. Acad. Sci. U. S. A..

[50] Teicher B.A., Fricker S.P. (2010). Clin. Cancer Res..

[51] Stellos K., Bigalke B., Langer H., Geisler T., Schad A., Kogel A., Pfaff F., Stakos D., Seizer P., Muller I., Htun P., Lindemann S., Gawaz M. (2009). Eur. Heart J..

[52] Hansen I.B., Ellingsen T., Hornung N., Poulsen J.H., Lottenburger T., Stengaard-Pedersen K. (2006). J. Rheumatol..

[53] Potter S.M., Dwyer R.M., Curran C.E., Hennessy E., Harrington K.A., Griffin D.G., Kerin M.J. (2009). Breast Cancer Res. Treat..

[54] Schutt R.C., Burdick M.D., Strieter R.M., Mehrad B., Keeley E.C. (2012). Stroke.

